# Malignant Glomus Tumor (Glomangiosarcoma) of Intestinal Ileum: A Rare Case Report

**DOI:** 10.1155/2013/305321

**Published:** 2013-04-08

**Authors:** Ahmed Abu-Zaid, Ayman Azzam, Tarek Amin, Shamayel Mohammed

**Affiliations:** ^1^College of Medicine, Alfaisal University, P.O. Box 50927, Riyadh 11533, Saudi Arabia; ^2^Department of Surgical Oncology, King Faisal Specialist Hospital and Research Center (KFSH&RC), P.O. Box 3354, Riyadh 11211, Saudi Arabia; ^3^Department of Pathology and Laboratory Medicine, King Faisal Specialist Hospital and Research Center (KFSH&RC), P.O. Box 3354, Riyadh 11211, Saudi Arabia

## Abstract

Glomus tumors are rare mesenchymal neoplastic lesions arising from glomus bodies that are involved in skin thermoregulation. They are mostly benign tumors, and malignant variants have been rarely reported. The subungual zones of fingers and toes are the most frequent sites of observation. Glomus tumors arising in visceral organs of the gastrointestinal tract are exceedingly rare. Stomach antrum and intestinal duodenum are the most frequent organs involved. No single case of glomus tumor involving intestinal ileum has been previously reported in the English medical literature. To the best of our knowledge, we report the first case of malignant glomus tumor (glomangiosarcoma) of intestinal ileum in a 29-year-old female patient who presented with a 1-month history of a tender pelvi-abdominal mass, constipation, vomiting, and melena. The intestinal ileum glomus tumor was resected, and histopathological diagnosis was consistent with glomangiosarcoma. A postoperative 6-month followup failed to show any evidence of tumor recurrence.

## 1. Introduction

Glomus tumors are mesenchymal neoplastic lesions arising from glomus bodies that are involved in skin thermoregulation [1, 2]. These neoplasms are extremely rare accounting for roughly 2% of all soft tissue neoplasms [3]. They mostly occur in the peripheral soft tissues with high tendencies to involve the dermal and subdermal subungual zones of fingers and toes [1, 2]. Glomus tumors barely take place in visceral organs, such as the gastrointestinal tract, where glomus bodies are scarcely present or even absent [4]. Among the very few reported cases of gastrointestinal glomus tumors, stomach antrum and duodenum were the most frequent regions involved [5]. The vast majority of reported cases demonstrated benign lesions. Malignant variants of glomus tumors (aka glomangiosarcomas) are exceedingly rare [6] and account for less than 1% of all glomus tumor cases [7]. Glomus tumors specifically involving the intestinal ileum are exceptionally uncommon with no single case previously reported in the English medical literature. To the best of our knowledge, we report the first case of glomangiosarcoma of the intestinal ileum.

## 2. Case Report

A 29-year-old female patient was referred to our hospital with a 1-month history of a pelvi-abdominal mass, constipation, vomiting, and melena. Upon presentation, the patient was anemic and had a tender palpable mass at the right lower quadrant. Laboratory investigations showed plasma hemoglobin (Hb) of 69 g/L (normal range values: 110–160 g/L) and plasma CA-125 of 85 U/mL (normal range values <35 U/mL). The patient was admitted and subjected to further investigations.

**Figure 1:**
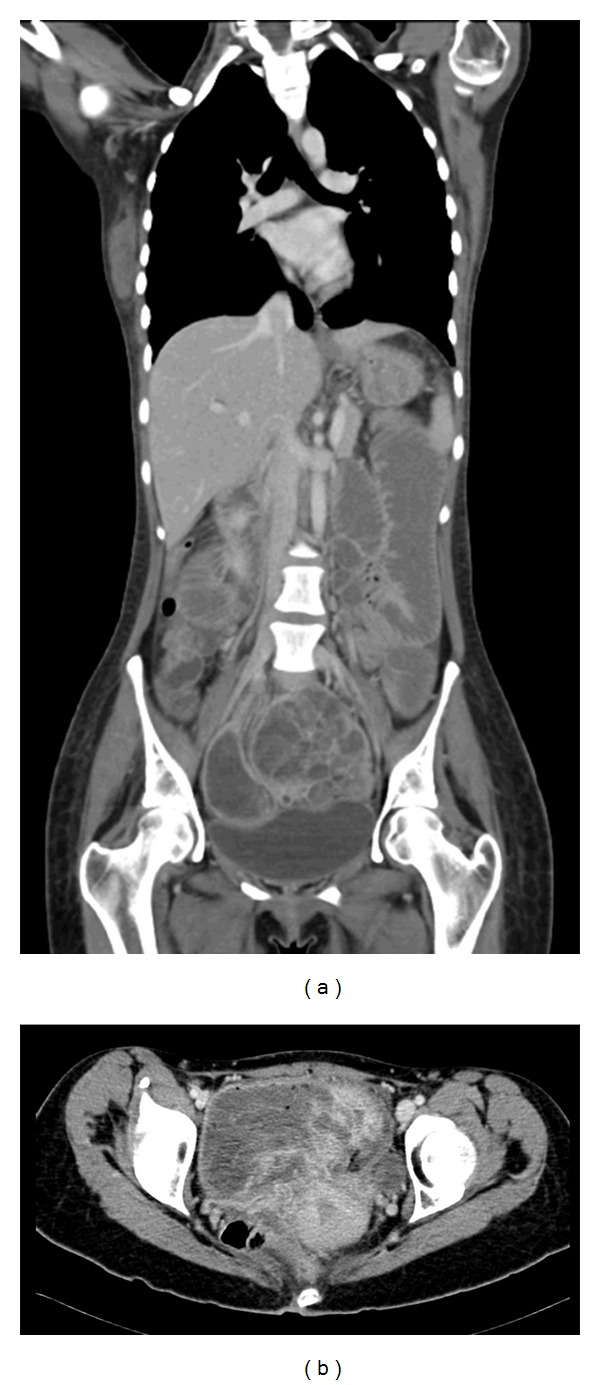
Computed tomography (CT) of the abdomen. (a) Coronal section and (b) axial section showing a huge 12.8 × 10.2 × 13.1 cm heterogeneous, aggressive-looking intraperitoneal pelvic mass lesion, most likely arising from intestinal ileum, with areas of lobulations, hemorrhagic cystic changes, gas locules, fluid-fluid levels, and proximal small bowel dilatation due to partial obstruction.

An abdominal X-ray was done and showed multiple dilated small bowel loops associated with air-fluid levels. The appearance was highly suggestive of partial intestinal obstruction. An enteroscopy was done and showed a huge submucosal mass near the intestinal ileum. Multiple biopsies were obtained, and histopathological features of a spindle cell neoplasm with smooth muscle cell differentiation were identified. An abdominal computed tomography (CT) scan showed a huge 12.8 × 10.2 × 13.1 cm heterogeneous, aggressive-looking intraperitoneal pelvic mass lesion, most likely arising from intestinal ileum, with areas of lobulations, hemorrhagic cystic changes, gas locules, fluid-fluid levels, and proximal small bowel dilatation due to partial obstruction (Figures 1(a) and 1(b)). Consequently, the patient was referred for surgical intervention.

The patient underwent resection of the intestinal ileum tumor and appendix with enteroenteric anastomosis (x2). Macroscopic and microscopic examinations of the appendix showed no pathological diagnosis. 

**Figure 2:**
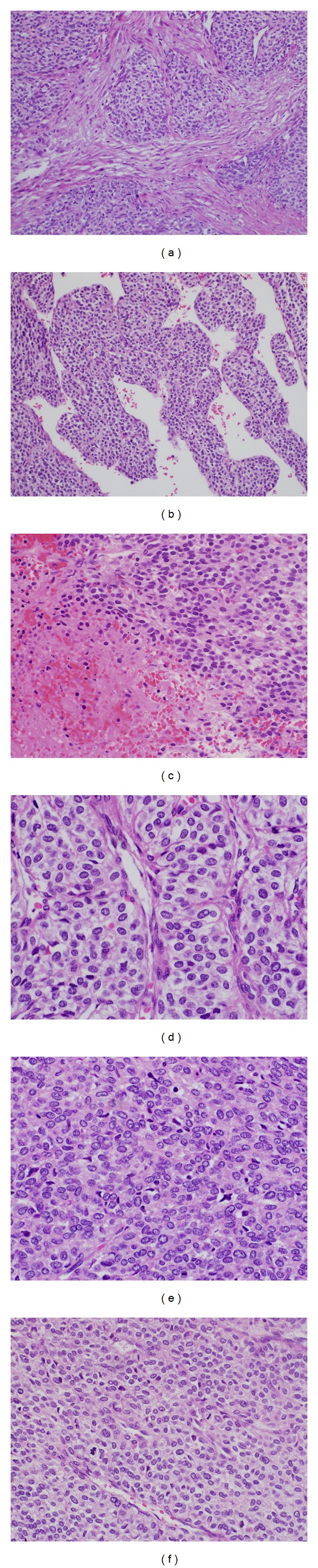
Histopathology of the excised intestinal ileum tumor. (a) Low power view showing multinodular growth. (b) Branching vascular channels lined by endothelial cells and interspersed by uniformly round to ovoid glomus cells forming nest, sheets, and trabeculae. (c) Area of geographic tumor cell necrosis. (d) Hypercellularity with distinct cell borders. (e) Areas of hypercellularity with mild atypia. (f) Focal spindled cells with scattered mitosis.

Grossly, the resected intestinal tumor of the ileum measured 14 cm in the maximum dimension with no definitive lymphovascular invasion was identified. Microscopically, the tumor was composed of multiple cellular nodules separated by streaks of smooth muscle cells or fibrous bands. The tumor ulcerated the overlying mucosa, involved mucosa, submucosa, and muscularis propria, and extended to the serosa. The tumor nodules showed a relatively solid pattern with pericytoma-like gaping capillary vessels. Cytoplasmic clearing was noted. The tumor cells showed sharply defined cell membranes and centrally located round, uniform nuclei with delicate chromatin and inconspicuous nucleoli with focal areas of spindled cells. Areas of coagulative necrosis were observed. Mitotic activity was approximately 4-5/50 high-power field (HPF) (Figures 2(a)–2(f)). 

**Figure 3:**
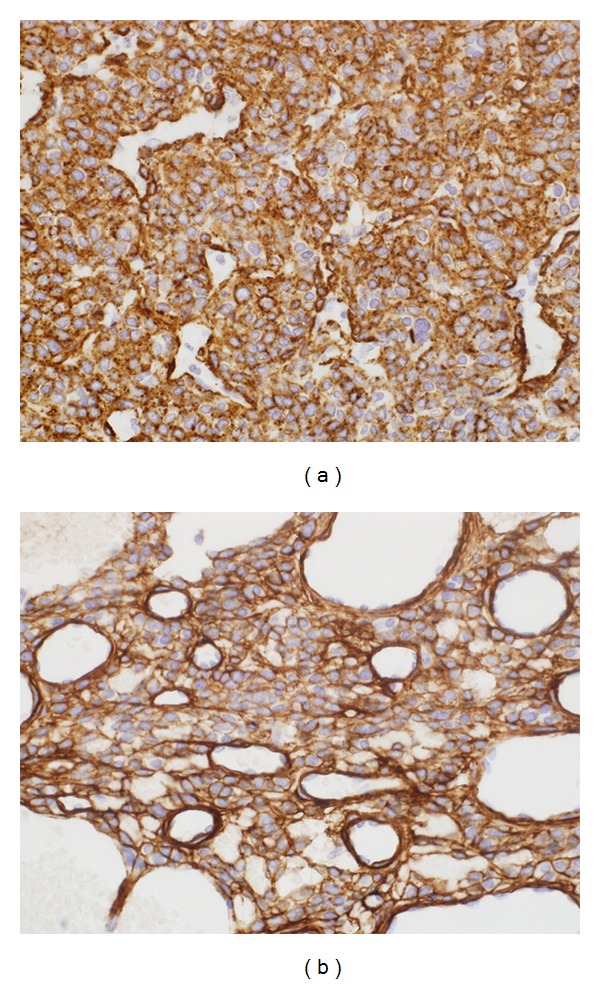
Immunohistochemistry of excised intestinal ileum tumor. (a) Tumor cells are positive for alpha smooth muscle actin (*α*-SMA). (b) Uniform pericellular type IV collagen expression.

Immunohistochemically, the intestinal ileum tumor cells were stained positive for alpha smooth muscle actin (*α*-SMA), h-caldesmon, and calponin. Pericellular net-like positivity for collagen type IV reticulin was also noted. The tumor cells were negative for CD117, CD34, cytokeratin, S100, desmin, human melanoma black-45 (HMB-45), chromogranin, and synaptophysin (Figures 3(a) and 3(b)). The histopathological diagnosis was consistent with malignant glomus tumor of the intestinal ileum.

A postoperative 6-month followup failed to show any evidence of tumor recurrence. Plasma hemoglobin (Hb) and CA-125 were within the normal ranges. The patient is doing fine and is to be followed up after 6 months.

## 3. Discussion

Glomus tumors are unusual soft tissue mesenchymal neoplasms composing 2% of all soft tissue neoplasms [3]. They originate from customized smooth muscle cells of the normal perivascular glomus bodies, which are modified arterio-venous anastomotic apparatuses involved in skin thermoregulation [1, 2]. They are frequently observed in the peripheral soft tissues, predominantly in the distal segments of extremities (i.e., subungual zones of fingers and toes) where glomus bodies are mostly abundant in the dermal and subdermal skin layers [1, 2]. Glomus tumors involving deep visceral organs are exceptionally unusual due to the relative deficiency or near absence of glomus bodies in these locations [4]. As such, diagnosis is often deferred or even missed.

Gastrointestinal glomus tumors are very uncommon, and the stomach antrum is the most frequent site of involvement followed by intestinal duodenum [5]. The majority of reported cases are of a benign nature, and malignant variants are considerably uncommon and almost vanishing [2], accounting for approximately 1% of all glomus tumor cases [7]. Moreover, gastrointestinal glomus tumors involving the intestinal ileum are exceedingly rare. In our search of the medical literature using PubMed, only a single case of intestinal ileum glomus tumor was reported in the Russian medical literature [8], whereas none was found in the English medical literature. To the best of our knowledge, this is the first reported case of a malignant glomus tumor (glomangiosarcoma) of intestinal ileum officially documented in the English medical literature.

Gastrointestinal glomus tumors present with a diversity of symptoms. In the setting of ulcerating overlying mucosa, upper (hemoptysis/hematemesis) or lower (hematochezia/melena) gastrointestinal tract bleeding is the main presenting symptom causing varying degrees of anemia with cardiopulmonary complications [2]. Other presenting symptoms may include nonspecific ulcer-like symptoms (retrosternal epigastric discomfort), nausea, and bilious vomiting secondary to bowel obstruction, while many other patients may remain symptom-free [9].

There are two forms of glomus tumors: solitary and multiple forms. Solitary forms are the most common accounting for roughly (90%) of all cases, occur most frequently in adults [10]. Multiple forms (i.e., multiple glomus tumor syndrome) are less common accounting for roughly (10%) of all cases and occur most frequently in children [10], and are believed to be inherited in an autosomal dominant manner with incomplete penetrance [11]. 

According to the microscopic morphology, glomus tumors can be divided into typical and atypical glomus tumors. Typical glomus tumors are histologically composed of glomus cells, blood vessels, and smooth muscle cells [11]. Histopathologically, typical glomus tumors can be further subdivided into solid tumors, glomangiomas, and glomangiomyomas based on the proportional abundance of round glomus cells, vascular smooth muscle cells, and spindle-shaped smooth muscle cells, respectively [12].

Several classifications of atypical glomus tumors have been proposed. In 1990, Gould et al. [6] proposed the following categorization of atypical glomus tumors: locally infiltrative glomus tumor (LIGT), glomangiosarcoma arising in a benign glomus tumor (GABG), and glomangiosarcoma arising de novo (GADN). LIGT has the typical glomus histological characteristics with an increased propensity to aggressively invade adjacent tissues. GABG and GADN are cytologically malignant lesions. The distinction between GABG and GADN is established by the presence or absence of a benign glomus tumor, respectively. GABG largely exhibits focal “spindling” of cells with cytologic neoplasia and hence is fairly simple to identify. Conversely, in GADN, the histopathological features suggestive of glomus tumors are often too minimal to be adequately recognized and distinguished from the round cell sarcomas [13]; and hence, establishing the correct diagnosis is fairly difficult and often missed.

In 2001, Folpe et al. [7] suggested the following classification of atypical and malignant glomus tumors: malignant glomus tumor (glomangiosarcoma), glomus tumor of uncertain malignant potential, symplastic glomus tumor, and glomangiomatosis. Folpe and colleagues [7] proposed the following criteria for classification of glomangiosarcomas: (a) deep tumor location and size more than 2 cm, (b) presence of atypical mitotic figures, or (c) combination of moderate-to-high nuclear grade and mitotic activity (5 mitoses/50 high-power fields). Although our patient had a deeply located, very large mass (14 cm in the maximum diameter) and increased mitotic activity (4-5 mitoses/50 high-power fields), interestingly, our patient did not develop distant metastasis. 

Generally, glomangiosarcomas are strongly stained positive for ki-67 (proliferation index marker), bcl-2 (antiapoptotic marker), and p53 (antiproliferative and apoptosis-inducing marker) than the benign glomus tumors [13, 14]. Glomangiosarcomas have an increased likelihood to recur locally [13]; and hence, long-term followup is greatly recommended. Regardless of bearing cytological characteristics of malignancy, glomangiosarcomas generally have a benign indolent clinical course and rarely metastasize, therefore providing excellent prognosis [2]. Nevertheless, malignant potential to aggressively invade distant organs is very predictable and cannot be ruled out; and therefore, long-standing followup is highly advised. Tumor location, mass, cellularity, nuclear atypia, spindle cell change, mitotic activities, atypical mitotic figures, necrosis, and angiolymphatic invasions have been fundamentally recognized as probable factors determining propensity for malignancy. Wide surgical excision is curative and remains the most effective treatment [2].

Gastrointestinal glomus tumors must be differentiated from the other closely cytology-related tumors such as gastrointestinal stromal tumors (GISTs), carcinoid tumors, hemangiopericytomas, paragangliomas, and lymphomas [1, 2, 15]. Immunohistochemical staining can be effectively used to facilitate a definitive diagnosis of a given neoplastic lesion. Gastrointestinal glomus tumors are nearly always positive for alpha-smooth muscle actin (*α*-SMA), calponin, h-caldesmon and vimentin [2, 7]. Gastrointestinal stromal tumors (GISTs) are almost always positive for CD117 (c-KIT) and very frequently (70%) positive for CD34, whereas gastrointestinal glomus tumors are persistently negative for CD117 and occasionally positive for CD34 [2]. Carcinoid tumors stain positively for keratin 18, chromogranin A, and synaptophysin. Gastrointestinal glomus tumors by no means express keratin 18 and chromogranin A proteins, whereas synaptophysin is only hardly ever expressed focally [3]. Hemangiopericytomas stain negative for alpha-smooth muscle actin (*α*-SMA) [2, 16], which is a satisfactory evidence to rule out a diagnosis of gastrointestinal glomus tumor. Paragangliomas stain substantially positive for chromogranin A and synaptophysin and exclusively positive for S-100 proteins, while gastrointestinal glomus tumors barely stain positive for S-100 proteins [2, 16]. Malignant lymphomas stain positive for CD20 and CD45 leukocyte-specific markers, both of which stain negative in gastrointestinal glomus tumors [2]. In short, immunohistochemical studies can simply and rapidly characterize the neoplastic profile of any given malignant lesion.

## 4. Conclusion

To the best of our knowledge, we report the first case of a malignant glomus tumor (glomangiosarcomas) of the intestinal ileum in the English medical literature. Glomus tumors of intestinal ileum are exceedingly rare but should be considered in the differential diagnosis in any patient presenting with a pelvi-abdominal mass. Moreover, histopathological examination of the excised lesion is fundamental for determining a definitive diagnosis. Glomangiosarcomas generally have a benign clinical course. Although glomangiosarcomas occasionally recur locally and rarely metastasize distally, long-term followup is greatly recommended as the recurrence, and malignant potentials cannot be excluded. Wide surgical resection is curative and remains the mainstay of treatment.
